# Comparing rural and urban mothers' awareness of self-harm and associated risk factors among high school students in Bangladesh

**DOI:** 10.1016/j.pmedr.2025.103127

**Published:** 2025-06-03

**Authors:** Mare-Cha, Md. Monir Hossain Shimul, Oiendrila Baroi, Salamat Khandker

**Affiliations:** Department of Public Health, Daffodil International University, Dhaka, Bangladesh

**Keywords:** Self-harm, Adolescents, Maternal awareness, Rural-urban differences, Bangladesh

## Abstract

**Objectives:**

Self-harm among adolescents is a growing public health concern, particularly in low- and middle-income countries. This study assessed maternal awareness of self-harm prevalence and associated risk factors among high school children in rural and urban Bangladesh.

**Methods:**

A descriptive cross-sectional study was conducted from July to October 2024 in 16 purposively selected schools (eight rural, eight urban) across three divisions of Bangladesh. A total of 480 randomly selected mothers of children in grades six–ten were interviewed using pre-tested, semi-structured questionnaires. Descriptive statistics summarized demographics and regression identified self-harm risk factors.

**Results:**

The awareness about children's self-harm was higher among urban mother's (30 %) compared to rural mother's (23.3 %). More urban mothers were concern of self-harm among high school going children as a significant issue (85.8 % vs. 35.8 %, *p* < 0.0001) and were more likely to seek additional information and resources (98.3 % vs. 75 %, *p* = 0.0002) to get a solution. Cutting and hitting were the common methods of self-harm in both settings, while starvation was more prevalent in urban areas (30.6 % vs. 10.7 %). Emotional distress, academic pressures, and social media were key triggers for urban adolescents, while rural children faced more family-related stressors. Urban parents were more inclined to seek professional help (22.2 % vs. 3.6 %, *p* < 0.05), while rural parents preferred community counseling.

**Conclusions:**

Significant rural-urban disparities exist among mother's awareness on self-harm and associated risk factors. Interventions to control emotional distress, academic pressure, and family related stressors are essential to address self-harm among adolescents in Bangladesh.

## Highlights

• Urban mothers had higher awareness of self-harm than rural mothers (30 % vs. 23.3 %).
• Cutting and hitting were common self-harm methods; starvation was more frequent in urban areas.
• Emotional distress, academic pressure, and social media influenced urban adolescents more.
• Rural parents preferred community counseling, while urban parents sought professional help.
• Interventions targeting emotional distress and family stressors are essential for prevention.

## 1 Introduction

Self-harm, is a growing public health concern, particularly among adolescents (Abdollahi et al., 2022; Al-Azhar Assiut Medical Journal, 2020). It manifests in various forms, including cutting, burning, scratching, hitting, and interfering with wound healing (Li et al., 2020; Alves et al., 2022). Meta-analyses of community-based studies report global adolescent self-harm prevalence ranging from 5 to 17 %, with higher rates in clinical samples (10–20 %) (McEvoy et al., 2023), the prevalence varies significantly across regions and demographic groups. Self-harm is closely associated with depression, anxiety, and post-traumatic stress (Sinha et al., 2021; Fariduzzaman et al., 2023).

Studies from India and Hong Kong have documented prevalence rates ranging from 3.2 % to 32.7 % (Doyle et al., 2015; Mind, n.d.). The majority of existing studies focus on university students, reporting a 17 % prevalence rate (Greydanus and Shek, 2009), leaving a significant gap in understanding the behaviors and risk factors among younger school-aged adolescents.

Adolescents aged 13–15 are vulnerable to self-harm due to developmental, social, and emotional challenges (Gillies et al., 2018). Academic pressure, social dynamics, and hormonal changes often contribute to anxiety and depression, leading some school-going children to use self-harm as a coping mechanism (Rasmussen et al., 2016). In Bangladesh, socio-cultural factors such as family structure, parental education, and economic disparities influence adolescent mental health (Geulayov et al., 2019). Urban adolescents face stress from social isolation, academic competition, and media exposure, while rural adolescents grapple with poverty, limited mental health resources, and traditional family expectations (Nawaz et al., 2021). Given that 40–60 % of suicides are preceded by self-harm, understanding these diverse risk factors is crucial for developing targeted interventions and preventive measures (ResearchGate, n.d.).

Although several studies have quantified self-harm prevalence among older adolescents and university students, very little is known about how parental (specifically maternal) awareness of self-harm risk factors varies by locality in Bangladesh. Given that parental recognition is key to early detection and intervention, a focused assessment of rural versus urban mothers' awareness can inform tailored community and school-based prevention programs. This descriptive cross-sectional study aims specifically to (1) compare rural and urban mothers' awareness of self-harm prevalence among high-school students, and (2) identify maternal recognition of key risk factors that could guide targeted preventive strategies in each setting. The study findings will provide evidence-based insights to policy formulation and develop targeted interventions for reducing self-harm behaviors among adolescents in Bangladesh.

## 2 Methodology

**Study Design and Settings**: This descriptive, cross-sectional study utilized a quantitative methodological approach to assess maternal awareness of self-harm and its associated risk factors among high school-going children in Bangladesh.

**Study Area and Population**: The study focused on the mothers of children attending classes six through 10 in the selected eight schools of Dhaka, four in Chittagong and four in Rangpur division of Bangladesh. School selection was based on population proportion, with Dhaka having four urban and four rural schools, while Chittagong and Rangpur had two urban and two rural schools each. The study population consisted of mothers whose children were enrolled in these schools. Participants were randomly selected from the pool of mothers whose children met the eligibility criteria.

**Sample Size and Sampling Technique:** Sample size was calculated using the formula n = z2pq/d2. Where *P* value was 0.17, d was 0.05 (Fariduzzaman et.al 2023) (Fariduzzaman et al., 2023) and the calculated sample size was 216. To account for a 10 % non-response rate and a design effect of 2 for cluster sampling, the target sample size was rounded up to 480.Sixteen schools were selected purposively. Mothers of six students were selected randomly from each class (Grades 6–10) ensuring diverse representation. Thus 30 mothers were chosen from each school ensuring comprehensive coverage across different educational levels.

**Inclusion and Exclusion Criteria**: Mothers of children studying in classes 6 to 10 who were willing to participate were included in the study. However, those who refused participation or had children outside this grade range were excluded.

**Data Collection Tools**: Data were collected using a pre-tested, semi-structured questionnaire comprising four sections: (1) demographic and socio-economic characteristics, (2) awareness of self-harm methods and prevention strategies, (3) perceived factors associated with self-harm and (4) general health and well-being of children. Self-harm was defined as any intentional non-fatal self-inflicted harm and was assessed via direct maternal report of specific behaviors (cutting, burning, starvation, etc.). Mothers were asked, ‘Has your child ever engaged in any of the following?’ with a checklist of seven methods. Mood swings and loneliness were each measured by single items adapted from the WHO STEPS instrument: ‘In the past month, how often did your child exhibit sudden mood changes (never/occasionally/frequently)?’ and ‘How often did your child feel isolated or lonely (never/occasionally/frequently)?’

The questionnaire was administered through face-to-face interviews. The clarity, relevance, and consistency in data collection was assessed during pre-test phase.

**Data Collection and Quality Control**: Data were collected between July and October 2024 from the study schools. Structured interviews were conducted by trained public health professionals. Quality control involved rigorous enumerator training, close supervision, and systematic review of completed questionnaires. A pre-test with a small sample refined the questionnaire, resolving ambiguities. Regular data verification addressed inconsistencies, ensuring reliability and integrity.

**Covariates:** Covariates included child-level, parent-level, and household-level factors, selected based on prior evidence linking them to self-harm risk. These comprised the child's age, sex, emotional and behavioral symptoms (e.g., mood swings, loneliness), maternal and paternal education, maternal and paternal occupation, family income, household structure (nuclear/joint), and residential setting (urban/rural). All variables were collected through face-to-face interviews using a pre-tested, semi-structured questionnaire. Behavioral indicators were based on maternal perceptions and coded as binary (present/absent) for analysis.

**Statistical Analysis:** First, descriptive statistics were used to summarize the demographic and socioeconomic characteristics of participants, stratified by urban and rural residence. Proportions and frequencies were calculated for categorical variables to identify rural–urban differences in maternal awareness and child behavior. Next, bivariate analyses (Chi-square tests) were conducted to examine associations between maternal awareness and each covariate. Following this, multivariable logistic regression analyses were performed to identify independent risk factors for self-harm among high school children, adjusting for all relevant covariates. Emotional and behavioral predictors (e.g., mood swings, loneliness) were prioritized based on theoretical relevance and prior significance in bivariate testing. Adjusted odds ratios (AORs) with 95 % confidence intervals (CIs) were calculated to quantify associations. All statistical analyses were conducted using SPSS version 23.

## 3 Results

Table 1 presents a comparative analysis of demographic and socioeconomic characteristics between rural and urban participants. Rural areas had a higher proportion of male children (57.5 %), whereas urban areas had more female children (60 %). The age distribution varied, with most rural children aged 14–16 years (42.5 %), while urban children were more evenly spread, with the highest proportion in the 11–13 years range (36.7 %). Mothers in rural areas were generally younger, with 22.5 % under 25 years, compared to only 7.5 % in urban areas. Additionally, 19.2 % of urban mothers were divorced or separated, compared to 6.7 % in rural areas. More rural families owned homes (85 %) than urban families (57.5 %), while urban households were more likely to be nuclear (93.3 %).

**Table 1:** Demographic and socioeconomic characteristics of mothers and their high school children in rural and urban areas of Bangladesh, July–October 2024.

Characteristic	Rural Area (*n* = 240) n (%)	Urban Area (*n* = 240) n (%)
*Sex of child*		
Male	138 (57.5)	96 (40.0)
Female	102 (42.5)	144 (60.0)
*Age of child*		
11–13 Years	82 (34.2)	89 (36.7)
14–16 Years	102 (42.5)	72 (30.0)
17–19 Years	60 (25.0)	80 (33.3)
*Age of the mother*		
< 25	54 (22.5)	18 (7.5)
26–35	100 (41.7)	52 (21.7)
36–45	86 (35.8)	162 (67.5)
>45	0 (0.0)	8 (3.3)
*Mother's present marital status*		
Living with husband	186 (77.5)	172 (71.7)
Widow	38 (15.8)	22 (9.1)
Divorced/Separated	16 (6.7)	46 (19.2)
*Living Place*		
Own house	204 (85)	138 (57.5)
Sublet	36 (15)	102 (42.5)
*Family Type*		
Nuclear Family	168 (70)	224 (93.3)
Joint Family	72 (30)	16 (6.7)
*Total number of family members*		
2–5	176 (73.3)	178 (76.7)
6–9	46 (19.2)	56 (23.3)
10–13	18 (7.5)	0 (0.0)
*Mother's Education level*		
Up to Primary	116 (48.3)	0 (0.0)
SSC	54 (22.5)	42 (17.5)
HSC	20 (8.3)	52 (21.7)
Degree/Hon's	16 (6.7)	78 (32.5)
Masters Hon's	34 (14.2)	68 (28.3)
*Father's education level*		
Up to Primary	86 (35.8)	0 (0.0)
SSC	40 (16.7)	52 (21.7)
HSC	32 (13.3)	26 (10.8)
Degree/Hon's	46 (19.2)	64 (26.7)
Masters Hon's	36 (15.0)	98 (40.8)
*Occupation of mother*		
Housewife	198 (82.5)	126 (52.5)
Government Service	16 (6.7)	22 (9.1)
Private Service	26 (10.8)	46 (19.2)
Business	0 (0.0)	18 (7.5)
Student	0 (0.0)	28 (11.7)
*Occupation of father*		
Government Service	32 (13.3)	76 (31.7)
Private Service	50 (20.8)	84 (35.0)
Business	84 (53.0)	58 (24.2)
Unemployed / Others	74 (30.8)	22 (9.1)
*Monthly family income*		
<35,000	92 (38.3)	26 (10.8)
36,000–47,000	44 (18.3)	54 (22.5)
48,000–59,000	34 (14.2)	68 (28.3)
60,000–75,000	22 (9.1)	42 (17.5)
>75,000	48 (20.0)	50 (20.8)

Education and income disparities were evident between rural and urban participants. No urban mothers had only a primary education, whereas 48.3 % of rural mothers did. Similarly, 40.8 % of urban fathers held a master's degree, compared to 15 % in rural areas. Most rural mothers were housewives (82.5 %), while urban mothers were more employed in private (19.2 %) or government (9.1 %) jobs. Rural fathers mainly ran businesses (53 %), whereas urban fathers worked in private (35 %) and government (31.7 %) sectors. Urban families reported higher incomes, with 28.3 % earning 48,000–59,000 BDT, compared to 14.2 % in rural areas.

Urban mothers demonstrated significantly higher awareness of self-harm among children, with 85.8 % recognizing it as a major issue compared to 35.8 % of rural mothers (95 % CI [−57.05, −42.95], Table 2). Similarly, 98.3 % of urban parents expressed interest in additional resources for prevention of self-harm compared to 75 % of rural parents (95 % CI [−28.56, −18.04]). Urban parents also exhibited a greater inclination toward structured interventions, with 25 % favoring parenting workshops (vs. 14.2 % in rural areas, 95 % CI [−18.98, −2.62]) and 21.7 % preferring support groups (vs. 7.5 %, 95 % CI [−21.95, −6.45]). In contrast, rural parents showed a higher preference for community counseling (31.6 % vs. 14.2 %, 95 % CI [9.41, 25.39]) and school-based programs (21.7 % vs. 8.3 %, 95 % CI [6.14, 20.66]).

**Table 2:** Awareness, Experiences, and Support Preferences Regarding Children's Self-Harm Among Rural and Urban Mothers in Bangladesh, July–October 2024.

Characteristic	Rural (*n* = 240) n (%)	Urban (*n* = 240) n (%)	Difference (95 % CI)
Recognition of self-harm as a significant issue among high school students.	86 (35.8)	206 (85.8)	−50.0 (−57.05, −42.95)
Experience of child engaging in self-harm.	56 (23.3)	72 (30.0)	−6.7 (−15.47, 2.07)
Confidence in managing child's self-harm behavior.	4 (1.6)	16 (6.7)	−5.1 (−8.72, −1.48)
Awareness of available support services.	0 (0.0)	0 (0.0)	–
Interest in receiving more information and resources on support systems.	180 (75.0)	236 (98.3)	−23.3 (−28.56, −18.04)
*Preferred types of support for parents dealing with a child who self-harms:*			
Parenting workshops	34 (14.2)	60 (25.0)	−10.8 (−18.98, −2.62)
Support groups for parents	18 (7.5)	52 (21.7)	−14.2 (−21.95, −6.45)
Access to mental health professionals	26 (10.8)	34 (14.2)	−3.4 (−10.53, 3.73)
School program on self-harm	52 (21.7)	20 (8.3)	13.4 (6.14, 20.66)
Information and resources on self-harm	34 (14.2)	40 (16.6)	−2.4 (−9.97, 5.17)
Community counseling	76 (31.6)	34 (14.2)	17.4 (9.41, 25.39)

Table 3 highlights the variations in self-harm methods. Urban parents were more likely to become aware of self-harm through visible physical signs (52.7 %) compared to rural parents (39.3 %). Cutting was the most commonly reported method in both groups, affecting 25 % of rural and 27.8 % of urban children. However, rural children more frequently engaged in hitting (28.6 % vs. 8.3 %, *p* < 0.05), whereas starvation as a method of self-harm was significantly more common among urban children (30.6 % vs. 10.7 %, *p* < 0.05). The frequency of self-harm also varied, with 13.9 % of urban children engaging in self-harm weekly compared to none in rural areas. Most self-harm incidents in urban settings occurred at home (63.8 %). Urban parents were more likely to seek professional help (22.2 % vs. 3.6 %, p < 0.05), whereas rural parents either ignored the behavior (35.7 %) or engaged their child in conversation (35.7 %).

**Table 3:** Reported Methods, Situational Triggers, and Parental Responses to Self-Harm Among High School Children as Reported by Mothers in Rural and Urban Bangladesh, July–October 2024.

Characteristic	Rural (*n* = 56) n (%)	Urban (*n* = 72) n (%)
*How did you become aware of child's self-harm?*		
Child told me	2 (3.6)	12 (16.7)
Noticed physical signs	22 (39.3)	38 (52.7)
Told by a teacher	4 (7.1)	4 (5.6)
Told by other children	28 (50.0)	18 (25)
*What methods of self-harm have you observed or been informed about?*		
Cutting	14 (25)	20 (27.8)
Burning	2 (3.6)	0 (0)
Hitting	16 (28.6)	6 (8.3)
Scratching or picking at skin	6 (10.7)	8 (11.1)
Pulling out hair	8 (14.3)	6 (8.3)
Ingesting harmful substances	4 (7.2)	10 (13.9)
Starvation	6 (10.7)	22 (30.6)
*If cutting, which parts of the body had your child chosen to cut?*		
Forearm	6 (42.9)	6 (30.0)
Wrist	6 (42.9)	8 (40.0)
Thigh	0 (0.0)	4 (20.0)
Chest	2 (14.2)	2 (10.0)
*How frequently does your child engage in self-harm?*		
Weekly	0 (0.0)	10 (13.9)
Monthly	12 (21.4)	10 (13.9)
Occasionally	18 (32.0)	22 (30.5)
Not sure	26 (46.3)	30 (41.7)
*Where does your child typically engage in self-harm?*		
At home	18 (32.1)	46 (63.8)
In private outside home and school	12 (21.4)	14 (19.4)
Not sure	26 (46.3)	14 (19.4)
*In what situations have you noticed your child engaging in self-harm?*		
After arguments	16 (28.6)	12 (16.7)
Following poor academic performance	4 (7.1)	18 (25)
Social issues (e.g., bullying, peer pressure)	12 (21.4)	14 (19.4)
Emotional distress (sadness, anxiety)	8 (14.3)	6 (8.3)
Family issues	12 (21.4)	4 (5.6)
Social media influence	0 (0.0)	14 (19.4)
Others	4 (7.1)	4 (5.6)
*How do you usually respond when you notice your child has self-harmed?*		
Talk to them	20 (35.7)	28 (38.9)
Seek professional help	2 (3.6)	16 (22.2)
Punish them	14 (25.0)	16 (22.2)
Ignore	20 (35.7)	12 (16.7)

Table 4 provides insight into the emotional and behavioral differences between rural and urban children. Rural children were more likely to be described as cheerful and energetic (74.2 % vs. 51.7 %, 95 % CI [10.51, 34.49]), while urban children exhibited higher rates of feeling down or uninterested (48.3 % vs. 25.8 %, 95 % CI [−34.49, −10.51]). Frequent mood swings were significantly more prevalent among urban children (62.5 % vs. 34.2 %, 95 % CI [−39.44, −17.16]), as were expressions of sadness or depression (52.5 % vs. 34.2 %, 95 % CI [−29.48, −7.12]). Feelings of isolation and loneliness were also more commonly reported by urban children (36.7 % vs. 21.7 %, 95 % CI [−26.03, −3.97]).

**Table 4:** Emotional and Behavioral Risk Factors for Self-Harm Among High School Children as Perceived by Mothers in Rural and Urban Bangladesh, July–October 2024.

Characteristic	Rural (n = 240) n (%)	Urban (n = 240) n (%)	Difference (95 % CI)
Child frequently appears cheerful and energetic.	178 (74.2)	130 (51.7)	22.5 (10.51, 34.49)
Child often seems down or uninterested in activities.	62 (25.8)	116 (48.3)	−22.5 (−34.49, −10.51)
Frequent mood swings observed in child.	82 (34.2)	150 (62.5)	−28.3 (−39.44, −17.16)
Expressions of sadness or depression by child.	82 (34.2)	126 (52.5)	−18.3 (−29.48, −7.12)
Recent significant changes in child's behavior or personality.	46 (19.2)	60 (25.0)	−5.8 (−14.77, 3.17)
Positive peer relationships at school.	185 (76.7)	160 (66.7)	10.0 (−0.85, 20.85)
Experiences of bullying or peer pressure.	123 (50.8)	136 (56.7)	−5.9 (−16.73, 4.93)
Feelings of isolation or loneliness in child.	52 (21.7)	88 (36.7)	−15.0 (−26.03, −3.97)
Presence of substance abuse issues within family.	42 (17.5)	60 (25.0)	−7.5 (−16.35, 1.35)

A multivariable logistic regression analysis was conducted to identify independent predictors of self-harm among high school students (Table 5). After adjusting for demographic and socioeconomic factors, it was found that children with frequent mood swings were 7.34 times more likely to engage in self-harm (OR = 7.34, 95 % CI [1.63, 33.03]). Additionally, children who expressed feelings of loneliness had a markedly increased risk, with an odds ratio of 98.88 (95 % CI [12.39, 789.26]). Interestingly, urban residency was found to be a protective factor, with urban children being 74.9 % less likely to engage in self-harm than their rural counterparts (OR = 0.251, 95 % CI [0.067, 0.942]).

**Table 5:** Multivariable Logistic Regression Analysis of Risk Factors for Self-Harm Among High School Children in Bangladesh Based on Maternal Report, July–October 2024.

Variable	Odds Ratio (OR)	95 % CI for OR
Mood Swings	7.34	(1.63, 33.03)
Loneliness	98.88	(12.39, 789.26)
Urban Residence	0.25	(0.07, 0.94)

ORs were adjusted for child's age, sex, family income, parental education, and family type (nuclear/joint).

## 4 Discussion

This study explored maternal awareness of self-harm among high school students in both rural and urban areas of Bangladesh, identifying key differences in recognition, response, and contributing factors. The findings revealed that urban mothers generally exhibited greater awareness and a stronger inclination toward seeking support services, while rural mothers were more inclined toward community-based solutions. The study also highlighted that adolescents in urban areas are more likely to experience emotional distress linked to academic pressures, digital media influence, and social isolation. In contrast, rural adolescents face distinct challenges, often shaped by familial stress and lower exposure to structured mental health resources. These patterns underscore the complex interplay between socio-demographic contexts and maternal perceptions of self-harm, emphasizing the need for tailored, context-sensitive interventions to effectively prevent and address adolescent self-harm.

The observed self-harm prevalence rates of 23.3 % in rural areas and 30 % in urban areas highlight the significant scale of this issue, with urban adolescents exhibiting higher rates. This trend aligns with existing literature from South Asia, where urbanization, academic stress, and digital exposure contribute to increased self-harm behaviors (Doyle et al., 2015; Mind, n.d.). Studies from India and other low- and middle-income countries (LMICs) similarly report higher self-harm prevalence in urban settings, suggesting the influence of socio-environmental stressors on adolescent mental health (Greydanus and Shek, 2009).

The distinct socio-demographic differences between rural and urban populations underscore the varying risk factors for self-harm. Rural children were more likely to come from joint families with lower parental educational attainment, while urban children predominantly lived in nuclear families with parents holding higher education levels (Geulayov et al., 2019; Nawaz et al., 2021). These structural differences could explain the greater emotional vulnerability among urban adolescents, who may experience increased pressure to succeed academically and socially. Similar findings have been reported in LMICs, where nuclear family structures and urbanization correlate with heightened psychological distress among adolescents (Mind, n.d.).

Cutting and hitting were the most commonly reported methods across both settings, consistent with global findings (Li et al., 2020; Alves et al., 2022). However, rural adolescents demonstrated a higher prevalence of hitting and burning as self-harm methods, whereas urban adolescents were more likely to engage in starvation (Geulayov et al., 2019; Quarshie et al., 2020; Epstein et al., 2020). These differences may reflect distinct socio-cultural norms, coping mechanisms, and access to harmful tools. The impact of emotional distress, family conflicts, and academic pressures on self-harm behaviors was evident in both groups, though urban adolescents were more affected by social media influences and performance anxiety. The role of digital exposure in exacerbating self-harm behaviors has been well-documented in prior studies, particularly in high-income countries where social media plays a critical role in shaping adolescent mental health (Fariduzzaman et al., 2023; Gillies et al., 2018).

Parental awareness and attitudes toward self-harm exhibited notable differences between rural and urban settings. Urban parents displayed greater recognition of self-harm as a major issue and showed more willingness to seek professional resources. In contrast, rural parents demonstrated lower awareness levels but expressed a preference for community-based interventions such as school-based counseling programs (Geulayov et al., 2019). These findings indicate the need for region-specific prevention strategies. Urban interventions should emphasize digital awareness campaigns and structured workshops for parents and educators, while rural initiatives may be more effective if embedded within community-driven programs and school-based counseling services (Rasmussen et al., 2016).

This study emphasized the interventions to control emotional distress, academic pressure, and family related stressors to address self-harm among adolescents in Bangladesh, that is consistent with previous study reports that schools, communities, and healthcare providers must work collaboratively to establish support systems that mitigate self-harm behaviors and enhance adolescent mental well-being (Nawaz et al., 2021; ResearchGate, n.d.).

The reliance on self-reported data may lead to underreporting or over reporting of self-harm behaviors, while its cross-sectional design limits the ability to establish causality between risk factors and self-harm. Additionally, the study focuses only on high school students, excluding younger adolescents and out-of-school youth. Selection bias may also be present despite efforts to ensure diverse socio-economic representation.

To address these gaps, future research should adopt longitudinal designs and include broader adolescent populations. Based on the findings, short-term recommendations include training teachers to identify self-harm, and expanding parental workshops and community counseling. Long-term strategies should involve policy interventions to mitigate systemic stressors like academic pressure and social media influence. Future research should also assess the effectiveness of these measures and explore additional risk factors, such as peer relationships and substance use, to promote adolescent mental health in Bangladesh.

## 5 Conclusion

This study underscores the urgent need for targeted interventions to address self-harm among adolescents in Bangladesh. The findings highlight the interplay of socio-demographic factors, emotional distress, and parental awareness in shaping self-harm behaviors. By addressing the research objectives, the study contributes valuable insights to inform school-based mental health programs, community counseling initiatives, and broader policy strategies. Future research should expand on these findings to develop comprehensive, evidence-based approaches aimed at promoting adolescent mental health and well-being.

## Availability of data and materials

The data supporting the conclusions of this article are included within the article.

## Human Ethics and Consent to Participate

Informed consent was obtained before data collection, ensuring voluntary participation with the option to withdraw anytime without consequences. Confidentiality and anonymity were strictly maintained. The study adhered to the ethical principles of the Declaration of Helsinki.

## CRediT authorship contribution statement

**Mare-Cha:** Writing – original draft, Methodology, Investigation, Formal analysis, Data curation, Conceptualization. **Md. Monir Hossain Shimul:** Writing – review & editing, Writing – original draft, Methodology, Formal analysis. **Oiendrila Baroi:** Writing – review & editing, Data curation. **Salamat Khandker:** Writing – review & editing, Resources, Methodology.

## Ethical Clearance

Ethical approval for the study was obtained from the Research Ethics Committee of the Faculty of Health and Life Sciences, Daffodil International University, Dhaka, Bangladesh (Approval Reference: FHLSREC/DIU/2024/SMIG-13) on June 23, 2024.

## Funding

This research did not receive any specific grant from funding agencies in the public, commercial, or not-for-profit sectors.

## Declaration of competing interest

The authors declare that they have no known competing financial interests or personal relationships that could have appeared to influence the work reported in this paper.

## Data Availability

The data supporting the conclusions of this article are included within the article.
